# Use and effectiveness of rituximab in children and young people with juvenile idiopathic arthritis in a cohort study in the United Kingdom

**DOI:** 10.1093/rheumatology/key306

**Published:** 2018-10-24

**Authors:** Lianne Kearsley-Fleet, Sunil Sampath, Liza J McCann, Eileen Baildam, Michael W Beresford, Rebecca Davies, Diederik De Cock, Helen E Foster, Taunton R Southwood, Wendy Thomson, Kimme L Hyrich

**Affiliations:** 1 Arthritis Research UK Centre for Epidemiology, University of Manchester, Manchester Academic Health Science Centre, Manchester, UK; 2 Clinical Academic Department of Paediatric Rheumatology, Alder Hey Children’s NHS Foundation Trust, UK; 3 Institute of Translational Medicine (Child Health), University of Liverpool, Liverpool, UK; 4 Musculoskeletal Research Group, Institute of Cellular Medicine, Newcastle University, Newcastle upon Tyne, UK; 5 Paediatric Rheumatology, Great North Children’s Hospital, Newcastle upon Tyne, UK; 6 Institute of Child Health, University of Birmingham and Birmingham Children’s Hospital, Birmingham, UK; 7 Arthritis Research UK Centre for Genetics and Genomics, Centre for Musculoskeletal Research, Faculty of Biologic, Medicine and Health, University of Manchester, Manchester, UK; 8 National Institute of Health Research Manchester Biomedical Research Centre, Manchester University NHS Foundation Trust, Manchester Academic Health Science Centre, Manchester, UK

**Keywords:** disease activity, JIA, observation study, registry, rituximab

## Abstract

**Objectives:**

Rituximab (RTX) may be a treatment option for children and young people with JIA, although it is not licensed for this indication. The aim of this study was to describe RTX use and outcomes among children with JIA.

**Methods:**

This analysis included all JIA patients within the UK Biologics for Children with Rheumatic Diseases study starting RTX. Disease activity was assessed at RTX start and at follow-up. The total number of courses each patient received was assessed. Serious infections and infusion reactions occurring following RTX were reported.

**Results:**

Forty-one JIA patients starting RTX were included, the majority with polyarthritis: polyarthritis RF negative [*n* = 14 (35%)], polyarthritis RF positive [*n* = 13 (33%)] and extended oligoarthritis [*n* = 9 (23%)]. Most were female (80%) with a median age of 15 years [interquartile range (IQR) 12–16] and a median disease duration of 9 years (IQR 5–11). The median improvement in the clinical Juvenile Arthritis Disease Activity Score (cJADAS; three-variable 71-joint JADAS) from RTX start was 9 units (*n* = 7; IQR −14–2). More than half reported more than one course of RTX. The median time between each course was 219 days (IQR 198–315). During follow-up, 17 (41%) patients reported switching to another biologic, including tocilizumab (*n* = 8), abatacept (*n* = 6) and TNF inhibitor (*n* = 3). Three patients (7%) reported a serious infection on RTX (rate of first serious infection 6.2/100 person-years). Four patients (10%) reported an infusion reaction.

**Conclusions:**

This real-world cohort of children with JIA, the majority with polyarticular or extended oligoarticular JIA, showed RTX may be an effective treatment option for children who do not respond to TNF inhibitor, with a low rate of serious infections on treatment.

**Rheumatology key messages**

- Rituximab is used in children with JIA who have not responded to other therapies.
- Rituximab may be an effective treatment option for many ILAR categories of JIA.
- The rate of serious infection and the number of infusion reactions in JIA were low.

## Introduction

JIA is the most common inflammatory rheumatic condition in childhood. It encompasses seven ILAR subtypes that differ in presentation and disease course [1]. Selective peripheral B cell depletion using rituximab (RTX) is a relatively recent advancement and an effective treatment approved for RA [2]. Although not licensed in JIA, it is considered a potential treatment option, particularly in RF-positive patients where TNF inhibitor (TNFi) treatment is ineffective or not tolerated [3]. In RA, each course of RTX consists of two 1000 mg i.v. infusions given 2 weeks apart, with further courses considered after 24 weeks [4]. The convenience of infrequent dosing, compared with other biologics, makes it an appealing therapeutic option in children.

There remains a question about which, if any, children may benefit from treatment with RTX. There are no clinical trial data for RTX in JIA. One observational study in 55 patients with severe JIA non-responsive to TNFi showed RTX (two courses of four weekly 375 mg/m^2^ i.v. infusions 24 weeks apart) to be effective, with 70% of patients achieving an ACR paediatric 70% response after 48 weeks [5]. The remaining evidence supporting RTX effectiveness consists of case reports or small case series, predominantly in adults with JIA [6–10]. There is evidence in RA that RTX may be more effective in RF-positive patients [11]. Whether this is also true for patients with JIA is currently unknown. The aim of this current analysis was to describe RTX use and effectiveness in a national cohort of children and young people with JIA.

## Methods

Participants were from the Biologics for Children with Rheumatic Diseases (BCRD) study [12], an ongoing national prospective observational cohort study established in 2010. The aim of the BCRD is to nationally monitor the effectiveness and safety of non-etanercept biologics in children and young people with JIA in routine clinical care in the UK. Children starting a non-etanercept biologic, regardless of prior biologic exposure, are recruited. Recruitment is recommended nationally [3], but not mandatory. Ethics approval for the BCRD was granted by the North West 7 Research Ethics Committee – Greater Manchester Central Ethics Committee. Patient or parent written informed consent was obtained as per the Declaration of Helsinki. Additional ethical approval to analyse these data was not required.

At registration into the BCRD, data are collected by the treating physician or affiliated clinical research nurse on patient demographics, disease features, disease activity including the JIA core outcome variables [13], past and current anti-rheumatic therapies and other medications. Follow-up data are collected at 6 months and 1 year, then annually, including any medication changes, current disease activity and occurrence of adverse events. When a patient starts a subsequent biologic after initial registration, additional data on disease activity at the start of the new therapy and after 6 months are requested in addition to the routine study follow-up data.

This analysis included all children from the BCRD who ever received RTX from 1 January 2010 through 31 January 2018. Disease activity measures recorded after approximately 6 months following the first RTX course were identified; a range of 4–12 months was allowed to account for variable timing of follow-ups. In addition, the three-variable 71-joint clinical Juvenile Arthritis Disease Activity Score (cJADAS) was calculated where data were available [14]. The change in core outcome variables and cJADAS was assessed regardless of whether patients had received additional courses of RTX or another biologic. The change in disease activity was assessed using the Wilcoxon matched-pairs signed-rank test. Doses of RTX received within 61 days of the previous dose were classified as part of the same course. The total number of RTX courses a patient received was determined. Further RTX courses received after an intervening alternative biologic drug were not included in the total (*n* = 1).

Patients were considered exposed (on drug) from each RTX infusion for 270 days or until the last study follow-up, 31 January 2018 (cut-off date), whichever came first. Exposure time for the first serious infection was censored at the time of the first serious infection. Rates of serious infection were calculated per 100 person-years of exposure [95% confidence intervals (CIs)]. Randomized controlled trials define infusion reactions in adults as any event occurring within 24 h of receiving RTX [2, 15]. The same definition was used in this analysis. Stata version 13 (StataCorp, College Station, TX, USA) was used to perform all analyses [16].

## Results

Of 834 biologic-treated patients, 41 (5%) had been exposed to RTX at some point—19 at the point of first registration and 22 as a subsequent biologic after registration. The reason for starting RTX was mostly inefficacy of previous therapy (62%) (Table 1). Overall, 80% were female, with a median age at the start of RTX therapy of 15 years [interquartile range (IQR) 12–16] and a median disease duration of 9 years (IQR 5–11). The majority of the patients had polyarthritis: polyarthritis RF negative [*n* = 14 (35%)], polyarthritis RF positive [*n* = 13 (33%)] or extended oligoarthritis [*n* = 9 (23%)]. Most patients (*n* = 40) had exposure to at least one biologic before starting RTX: 29% exposed to one, 39% exposed to two and 29% exposed to at least three. All patients with prior biologic exposure had received TNFi, 23% tocilizumab and 20% abatacept. Only 34% were on concomitant DMARD therapy, predominantly methotrexate (Table 1).

**Table 1:** Baseline characteristics of 41 patients with JIA at the start of RTX therapy

Characteristics	RTX patients
Total number of patients	41
Female, *n* (%)	33 (80)
Age, median (IQR), years	15 (12–16)
Disease duration, median (IQR), years^a^	9 (5–11)
ILAR^a^*n* (%)	
Systemic	–
Oligoarticular, persistent	2 (5)
Oligoarticular, extended	9 (23)
Polyarticular RF negative	14 (35)
Polyarticular RF positive	13 (33)
Psoriatic	1 (3)
Enthesitis related	1 (3)
Undifferentiated	–
Previous biologic exposure	40 (98)
Number of previous biologics, *n* (%)	
0	1 (2)
1	12 (29)
2	16 (39)
≥3	12 (29)
If yes, which class of biologic, *n* (%)	
TNFi	40 (100)
IL-6 pathway inhibitor (tocilizumab)	9 (23)
T cell co-stimulation blocker (abatacept)	8 (20)
Concurrent DMARDs	14 (34)
Ever glucocorticoids	40 (98)
RTX start reason^b^, *n* (%)	
Inadequate response/inefficacy to previous drug	24 (62)
Intolerance/adverse events on previous drug	5 (13)
Intolerance and inadequate response	6 (15)
Adherence issues with prior drug(s)	4 (10)

a Data missing in one patient.

b Data missing in two patients.

Twenty-nine patients (71%) had disease activity data available at baseline and follow-up. At the start of RTX the median active joint count was 3 (IQR 1–7), the median Childhood HAQ score was 1.2 (IQR 0.1–1.9) and the median cJADAS was 15 (IQR 8–18) (Table 2). At follow-up, disease activity had improved; the median change in active joint count was −1 (IQR −4 to 0; *P* < 0.05) and the median change in cJADAS was −9 units (IQR −14 to 2), although data availability on some variables were limited. Of these patients, one-quarter (*n* = 7) had received additional RTX courses and four patients had received another biologic prior to the follow-up assessment.

**Table 2:** Disease activity data at the start of RTX therapy and at the first follow-up (*N* = 29)

	Patients, *n*	Baseline, median (IQR)	Follow-up^a^, median (IQR)	Change from baseline, median (IQR)
Disease activity				
Active joint count (71-joint count)	23	3 (1–7)	1 (0–3)	−1 (−4–0)^b^
Limited joint count (71-joint count)	23	3 (2–8)	2 (0–4)	−2 (−3–0)^b^
Physician global (10 cm VAS)	9	5 (3–6)	2 (1–3)	−3 (−5–0)^b^
Patient well-being (10 cm VAS)	12	5 (3–7)	4 (2–5)	−2 (−2–0)
Childhood HAQ (0–3)	12	1.2 (0.1–1.9)	1.1 (0.2–1.6)	0.1 (−0.7–0.1)
Pain VAS (10 cm VAS)	11	5 (2–7)	4 (2–6)	−1 (−3–1)
ESR (mm/h)	27	15 (5–31)	8 (3–22)	−2 (−14–0)^b^
CRP (mg/l)	28	4 (4–7)	4 (4–10)	0 (−4–0)
cJADAS	7	15 (8–18)	5 (1–10)	−9 (−14–2)

a Follow-up ranged from 3–9 months. If more than one follow-up was available, the one closest to 6 months was selected. Of the 29 patients included, 18 were assessed after the first RTX course, four after a subsequent biologic therapy and seven after at least one subsequent RTX course.

b *P* < 0.05.

VAS: visual analogue scale.

Of the patients with dose data available (*n* = 34), the median RTX dose at the start of therapy was 1000 mg (IQR 900–1000). The majority of patients [*n* = 36 (88%)] received two doses of RTX in each course, of which 58% (*n* = 21) received 1000 mg for both doses. More than half of the patients reported more than one course of RTX: 17 (41%) had one, 12 (29%) had two, 7 (17%) had three and 5 (12%) had four or more. The median number of days between each course was 219 (IQR 198–315). Thirty-eight patients had follow-up recorded in the BCRD following their last recorded dose of RTX, with a median of 177 days (IQR 109–398). Forty-five per cent of those (*n* = 17) reported switching from RTX to another biologic, predominantly due to inefficacy (67%), after a median time of 178 days (IQR 133–306). Eight patients started tocilizumab (47%), six patients started abatacept (35%) and three patients started TNFi (18%). Eight of these patients switched after only one RTX course (47%), four after two (24%) and five after three or more courses (29%).

There was a total available exposure time on RTX therapy of 51 person-years. Three (7%) patients experienced a serious infection during this time; the rate of first serious infections was 6.2 per 100 person-years (95% CI 2.0, 19). One patient experienced a further infection on RTX; the rate of all serious infections was 7.8 per 100 person-years (95% CI 2.9, 21). All serious infections occurred within a range of 14–115 days following the most recent RTX infusion. Four (10%) patients experienced an infusion reaction. In all cases these reactions were mild, with no patient experiencing an anaphylactic response.

## Discussion

This is one of the largest observational studies describing RTX use and effectiveness in children and young people with JIA. Since 2010, 5% of patients in this national cohort have been exposed to RTX. As per the current 2015 National Health Service England guidelines for biologic therapies in the treatment of JIA, the majority of these patients started RTX therapy after failure of a TNFi [3]. However, despite these guidelines suggesting RTX be considered for RF-positive polyarthritis, based on evidence from RA studies [11], this ILAR category represented only one-third of patients treated. Unfortunately, within the current study, the total number of patients with polyarthritis was too small to form any conclusions regarding differential benefits by antibody status.

The majority of patients reported received two 1000 mg doses of RTX within each course, in accordance with European licencing indications for adults with RA [4]. Data were unavailable on body surface dose given, only data on the dose received was available. During the available follow-up, more than half the patients received at least two courses of RTX, with a median interval between each course of ∼7 months. Again, this is in line with the European guidelines for adults with RA and suggests that a benefit was received following the first course in many patients. A limitation of this study is that the reason patients received additional doses were unknown (whether due to worsening disease symptoms or a planned re-treatment).

Only one-third of patients reported receiving concomitant DMARD therapy when starting RTX treatment. The reasons for this low proportion are unknown. Overall, disease activity across a wide range of core outcome variables improved following RTX treatment. However, within the period of follow-up available, almost half of the patients reported switching to another biologic therapy, most following only one or two courses, suggesting that RTX, while effective in some patients, was not considered effective in others.

The rate of first serious infection in this analysis was slightly higher compared with rates reported in children and young people with JIA treated with first-line etanercept (6.2 *vs* 4.8 per 100 person-years), although the 95% CIs do overlap [17]. It is possible that, due to the second-line nature of the therapy, the patients starting RTX were fundamentally different, i.e. older (median age 15 *vs* 11 years) with longer disease duration (9 *vs* 3 years). Thus they may have more comorbidities, higher total steroid exposure or poorer disease activity, function or well-being compared with patients starting first-line etanercept. The rate of serious infection observed in the current analysis was similar to those seen in RA in both a randomized controlled trial (RCT) [2] and a cohort study [18]. In addition, four (10%) patients experienced an infusion reaction within 24 h of a RTX infusion. Infusion reactions are common in adults with RA receiving RTX and may potentially be life threatening [19]. Two previous RCTs of adults with RA found 29–38% of RTX-treated patients experienced an infusion reaction, compared with 18–23% of the placebo-treated patients [2, 15]. However, reporting of infusion reactions in the current study required the clinician to perceive that it was serious enough to record in the rheumatology record (in addition to the hospital record of the infusion itself) and also report to the study, which is likely to explain the differences between the two study designs.

The BCRD study is the UK’s largest cohort of JIA patients starting a non-etanercept biologic therapy. Registration into the study is highly encouraged [3] and therefore the numbers reported in this analysis are likely to represent a majority of RTX-treated patients in the UK. However, JIA is a rare disease with ∼20% of patients starting a biologic therapy [20], consequently the proportion of patients who may be considered for RTX will be even lower. As disease activity at the start of RTX therapy in patients already registered had to be additionally requested, rather than the mandatory nature of those newly registering, there were missing data. The limited patient numbers and amount of missing data within the dataset means that all patients were assessed at follow-up, regardless of additional treatment, and factors associated with good response could not be investigated. Multiple imputation, a method usually applied to account for missing data, could not be used on such a small number of patients.

In this real-world cohort of children and young people with JIA being treated with non-etanercept biologic therapy, ∼5% of patients across many ILAR categories have reported exposure to RTX therapy. Dosing was consistent with current guidelines for use in adults with RA, as no paediatric JIA dose has been studied. More than half of the patients report using RTX for more than one course, indicating potential benefit, with similar improvements in disease activity measures observed. These data are reassuring, but ultimately patients with JIA deserve a clinical trial of RTX therapy to establish clear efficacy and safety of the therapy, as well as indicating in which ILAR categories RTX therapy may be most effective. As patients continue to be followed within the BCRD study, real-world effectiveness can continue to be assessed.
